# Peroneus brevis split tear – A challenging diagnosis: A pictorial review of magnetic resonance and ultrasound imaging. Part 1. Anatomical basis and clinical insights

**DOI:** 10.1016/j.ejro.2024.100633

**Published:** 2025-01-08

**Authors:** Katarzyna Bokwa-Dąbrowska, Rafał Zych, Dan Mocanu, Michael Huuskonen, Dawid Dziedzic, Pawel Szaro

**Affiliations:** a Department of Radiology, Institute of Clinical Sciences, Sahlgrenska Academy, University of Gothenburg, Gothenburg, Sweden; b Department of Musculoskeletal Radiology, Sahlgrenska University Hospital, Gothenburg, Sweden; c Department of Clinical and Descriptive Anatomy, Medical University of Warsaw, Poland

**Keywords:** Tendon, Anatomy, Imaging, Ultrasound, Magnetic resonance imaging

## Abstract

Diagnosing peroneus brevis split tears is a significant challenge, as many cases are missed both clinically and on imaging. Anatomical variations within the superior peroneal tunnel can contribute to peroneus brevis split tears or instability of the peroneal tendons. However, determining which anatomical variations predispose patients to these injuries remains challenging due to conflicting data in the literature. In this review, we present the current understanding of the role of anatomical variants in the development of peroneus brevis split tears. Many studies emphasize the significance of the retromalleolar groove and retromalleolar tubercle, the impact of a low-lying muscle belly, and the presence of accessory muscles within the superior peroneal tunnel as contributors to peroneal pathology. Hypertrophy of the peroneal tubercle or post-traumatic irregularities in the surface of the retromalleolar groove can accelerate degenerative changes in the peroneal tendons, potentially leading to peroneus brevis split tears. The topographic anatomy of the superior peroneal tunnel is essential for systematically performing ultrasound and interpreting magnetic resonance imaging of the ankle. The first part of this review focuses on the anatomical foundations of imaging diagnostics for peroneus brevis pathology. In the second part, we will examine the radiological spectrum of peroneal tendon injuries, offering a framework to enhance diagnostic confidence in this frequently underdiagnosed pathology.

## Highlights

• Low-lying peroneus brevis may increase risk of peroneal split tears.
• Accessory muscles in the superior peroneal tunnel may cause overcrowding and tendon compression.
• Retromalleolar groove shape may affect the stability and function of peroneal tendons.
• Fibular misalignment can lead to friction and the peroneus brevis split.
• Hypertrophied peroneal tubercle, accelerate peroneus brevis degeneration and split development.

## 1 Clinical anatomy

### 1.1 Topography and normal anatomy

**Fig. 1:**
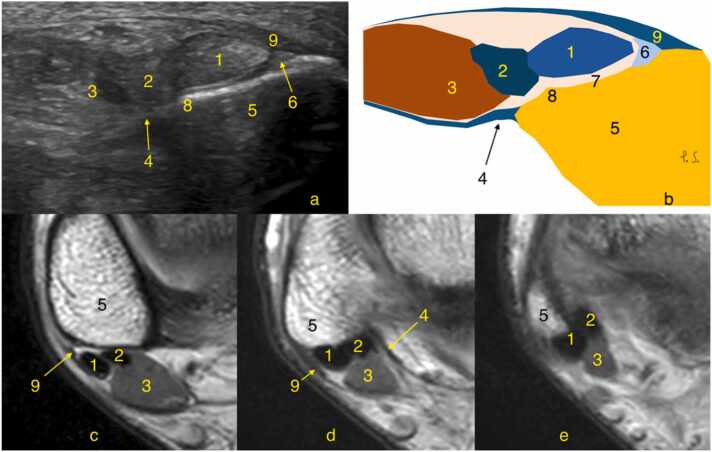
Cross-sectional images at the level of the lateral malleolus showing normal structures. Ultrasound (a), schematic diagram (b), magnetic resonance, proton density weighted (c, d, e). 1- Peroneus longus, 2- Peroneus brevis (tendon), 3- Peroneus brevis (muscle), 4- Crural fascia, 5- Fibula, 6- Fibrocartilage ridge, 7- Retromalleolar groove, 8- Retromalleolar tubercle, 9- superior peroneal retinaculum.

The peroneal tendons are the primary evertors of the foot, act as dynamic stabilizers[1], and play role in ankle proprioception[2]. At the level of the ankle joint, the peroneal tendons are located posterior to the lateral malleolus in the retromalleolar groove (sulcus malleolaris) (Fig. 1).

The position of the tendons in the peroneal groove depends on the position of the ankle and the load. The position of the tendons may also depend on the level of the cross-section. Just below the lateral malleolus, the peroneus brevis runs toward the base of the fifth metatarsal, while the peroneus longus heads toward the base of the first metatarsal. According to our observations, the crossing of these tendons typically occurs just below the lateral malleolus or, in some cases, at its level. In cases where the peroneus brevis is positioned medially, the crossing of the tendons may occur slightly higher or closer to the lateral malleolus. In a resting position, when the ankle joint is neither flexed nor extended, the peroneus longus is positioned slightly laterally, covering ca 2/3 of the peroneus brevis from behind. According to our unpublished observations, there are individual variations in the positioning of the peroneal tendons during ankle movements, making it advisable to compare with the opposite side. We also observe that in asymptomatic sides, there can be an appearance like subluxation. In our opinion, it can be challenging to definitively distinguish between a normal variant and pathology in borderline cases. Unfortunately, there is a lack of studies on the dynamic variability of the peroneal tendons in healthy persons. Based on our observations, during dorsiflexion, the peroneus brevis slides beneath the peroneus longus. In plantar flexion, the peroneal tendons remain in the same position as at rest.

**Fig. 2:**
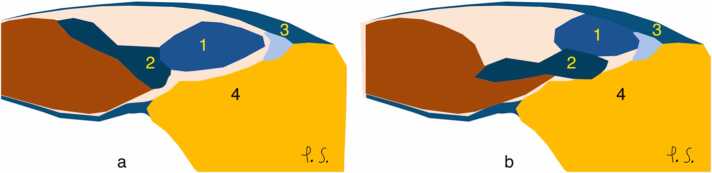
Normal change in the position of the peroneal tendons between the neutral position (a) and dorsiflexion with eversion (b) according to our observations. 1- Peroneus longus, 2- Peroneus brevis, 3- Superior peroneal retinaculum, 4- Fibula.

In the neutral (anatomical) position, the peroneus brevis tendon is typically located in the medial part of the malleolar groove, medial to the peroneal tubercle. In transverse cross section a normal peroneus brevis tendon appears crescent-shaped, with slightly thicker medial and lateral edges or oval depending on anatomical variations (Fig. 2). The boomerang shape of the peroneus brevis tendon occurs when the central part of the tendon is thinner[3], which should not be confused with the "boomerang sign" sign of split tear. On a transverse cross-section, the split fragments of the peroneus brevis wrap around the peroneus longus, which insinuates into the gap formed by the split. This appearance is referred to as the "boomerang sign"[4] discussed more in the second part of the review.

**Fig. 3:**
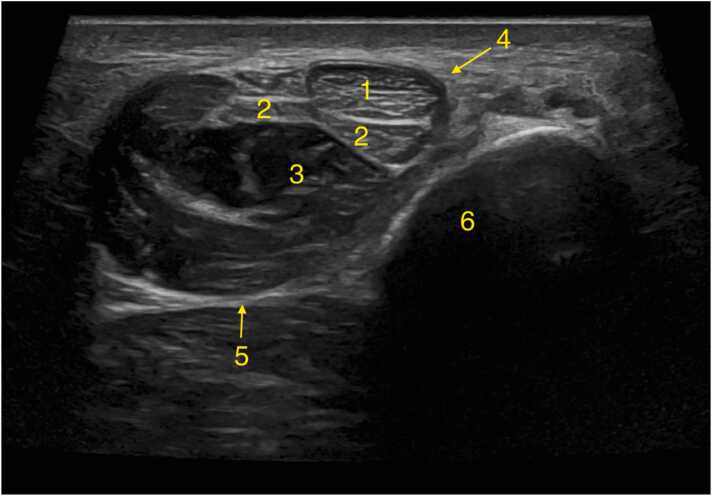
Ultrasound transverse section of the peroneal tendons at the level of the lateral malleolus, slightly superior to the superior peroneal tunnel. The image reveals normal variation of the peroneus brevis tendon with central flattening, accompanied by slight thickening in the medial and lateral regions. 1- Peroneus longus, 2- Peroneus brevis (tendon), 3- Peroneus brevis (muscle), 4- Superior peroneal retinaculum, 5- Crural fascia, 6- Fibula.

**Fig. 4:**
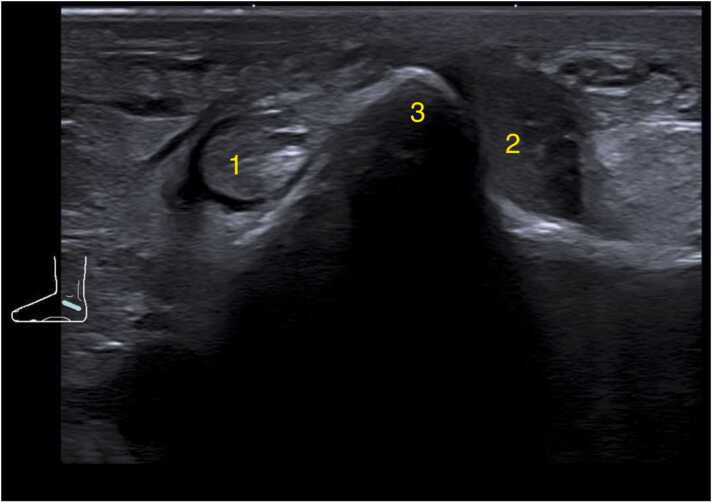
Ultrasound oblique cross-section at the level of the peroneal tubercle. The peroneus brevis tendon runs over the tubercle, while the peroneus longus tendon passes underneath it. 1- Peroneus longus, 2- Peroneus brevis with a small amount of fluid in the synovial tendon sheath, 3- Peroneal tubercle.

The peroneus longus tendon is larger and has an oval shape in cross-section (Fig. 3). Up to the level of the lateral malleolus, the tendons run vertically. As the tendons pass on the retromalleolar groove, beneath the superior peroneal retinaculum and superficial to the calcaneofibular ligament [5]. As they pass the lateral malleolus, the peroneus brevis tendon runs to the tuberosity of the fifth metatarsal bone, while the peroneus longus tendon moves below the peroneal tubercle of the calcaneus (Fig. 4).

The peroneus brevis attaches to the tuberosity of the fifth metatarsal. The peroneus longus tendon passes through a fibro-osseous tunnel beneath the cuboid bone (cuboid tunnel) and attaches to the plantar surfaces of the first metatarsal and medial cuneiform bones, adjacent to the insertion of the tibialis anterior tendon[6].

### 1.2 Superior peroneal retinaculum

The superior peroneal retinaculum originates from the posterior border of the retromalleolar groove and the tip of the lateral malleolus. Distally, the superior peroneal retinaculum interconnects with the paratenon of the Achilles tendon and the adjacent part of the lateral calcaneus[7]. The superior peroneal retinaculum is thickening of the crural fascia and interconnects with neighboring structures. These interconnections may provide an anatomical basis for explaining the concomitant injuries of multiple structures, such as the superior peroneal retinaculum and anterior talofibular ligament[7]. Preserving the integrity of the retinaculum is essential to prevent the subluxation or dislocation of the peroneal tendons.

### 1.3 Superior peroneal tunnel

#### 1.3.1 Limitations

**Fig. 5:**
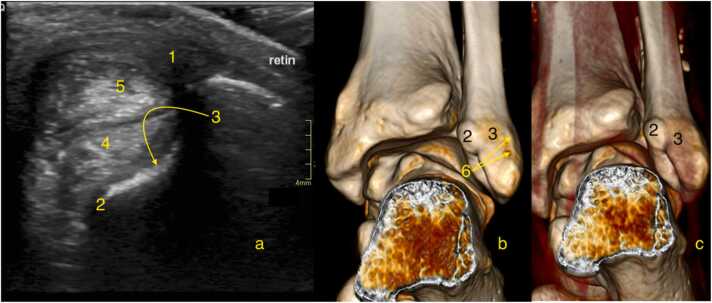
The retromalleolar groove. (A) Ultrasonography, (B, C) 3D reconstruction from computed tomography. 1- Superior peroneal retinaculum, 2- Retromalleolar tubercle, 3- Retromalleolar groove, 4- Peroneus brevis (tendon), 5- Peroneus longus, 6- Retromalleolar ridge. Retin - an abbreviation for the superior peroneal retinaculum. A subtle imprint of the peroneus tendons can be observed at the level of the posterior surface of the lateral malleolus, where the number 3 is visible, and slightly above it.

The superior peroneal tunnel is a fibro-osseous tunnel which has an oval shape in transverse cross-section. The tunnel is bordered anteriorly by the malleolar groove[8]. The groove is oriented obliquely and is bordered superiorly and medially by the retromalleolar tubercle, and inferolaterally by the retromalleolar ridge[8] (Fig. 1, Fig. 2, Fig. 5). In many cases, an imprint of the peroneal tendons can be seen on the posterior contour of the lateral malleolus, slightly superior to the malleolar groove (Fig. 5).

On the lateral border of the retromalleolar groove (Fig. 5) a thin fibrocartilage ridge is located (Fig. 1). It appears triangular on cross-sections. This fibrocartilage ridge deepens the groove in much the same way that the labrum deepens the sockets of the shoulder and hip joints. During forced dorsiflexion, especially when the foot is inverted or everted, the peroneal muscles can contract with enough force to overcome the superior retinaculum, potentially causing a temporary lateral peroneal dislocation.

The posterior-lateral boundary of the superior peroneal tunnel is formed by the superior peroneal retinaculum, while the anterior-medial boundary is an extension of the crural fascia called here posterior intermuscular septum[8], Fig. 1. The calcaneofibular ligament limits the inferior aperture of the tunnel on the medial side[9].

#### 1.3.2 Content

The superior peroneal tunnel houses the peroneus brevis and longus tendons, encased within a common synovial sheath that extends from approximately 4 cm proximal to 1 cm distal to the lateral malleolus[10]. The common synovial sheath is constricted at the level of the superior peroneal retinaculum. Typically, a larger amount of fluid is visible below this level, which in practice is just below the lateral malleolus, rather than at the level of the retinaculum.

In anatomical variations, as an additional tendon or a low-lying muscle, increased pressure in the superior peroneal tunnel may occur, leading to pain and the potential development of peroneal tendon pathology.

Recent studies have identified the accessory deep peroneal nerve as a consistently present structure, not an anatomical variant as thought before[11]. Originating from the superficial peroneal nerve, it travels deep to the peroneus longus along the posterior border of the peroneus brevis, possibly passing through the superior peroneal tunnel before winding around the lateral malleolus to reach the foot. This nerve provides motor and sensory innervation to the lateral part of the leg and ankle, including branches to the peroneus brevis, and occasionally to the peroneus longus, extensor digitorum brevis[11].

## 2 Clinical important anatomical variants

Morphological changes in the retromalleolar fibular groove, hypertrophy of the peroneal tubercle, the presence of accessory tendons, and a low-lying peroneus brevis muscle belly[1], [5], [12], [13], [14].

### 2.1 Morphology of the lateral malleolus

The retromalleolar groove may have convex, flat, or concave forms; however, most studies indicate that the convex groove is the most common, occurring in approximately 70–80 % of cases[15], [16], [17]. In most of cases, an imprint of the peroneal tendons is observed. Although the groove depth is usually shallow and difficult to measure, it occasionally reaches 2–3 mm[18]. The mean width of the malleolar groove is about 9 mm however it may vary dependent on the form[18]. The malleolar groove is oriented posteriorly and slightly laterally, however the version is variable[18]. It is unclear if the form of the malleolar groove may impact the split tear or stability of peroneal tendons. One study found no significant relationship between peroneal instability and the shape of the groove[17], while another study suggested that a flat or convex groove may be associated with peroneal tendon luxation. Most studies that focus only on the shape of the malleolar groove using computed tomography or dry bone specimens may underestimate its depth, as the absence of the fibrocartilaginous ridge makes the groove appear artificially shallow. The depth of the groove may be enhanced by the presence of the fibrocartilaginous ridge[15], [19].

### 2.2 Retromalleolar tubercle

The consistently present retromalleolar tubercle normally lies beneath the peroneus brevis tendon without projecting into superior peroneal tunnel. Post-traumatic changes, along with internal misalignment and rotation of the fibula can cause the retromalleolar tubercle to protrude into the tunnel, leading to tendon friction and possible split tear. There are reported cases where peroneus brevis splits occurred over a prominent retromalleolar tubercle[8].

### 2.3 Low lying peroneus brevis muscle belly

**Fig. 6:**
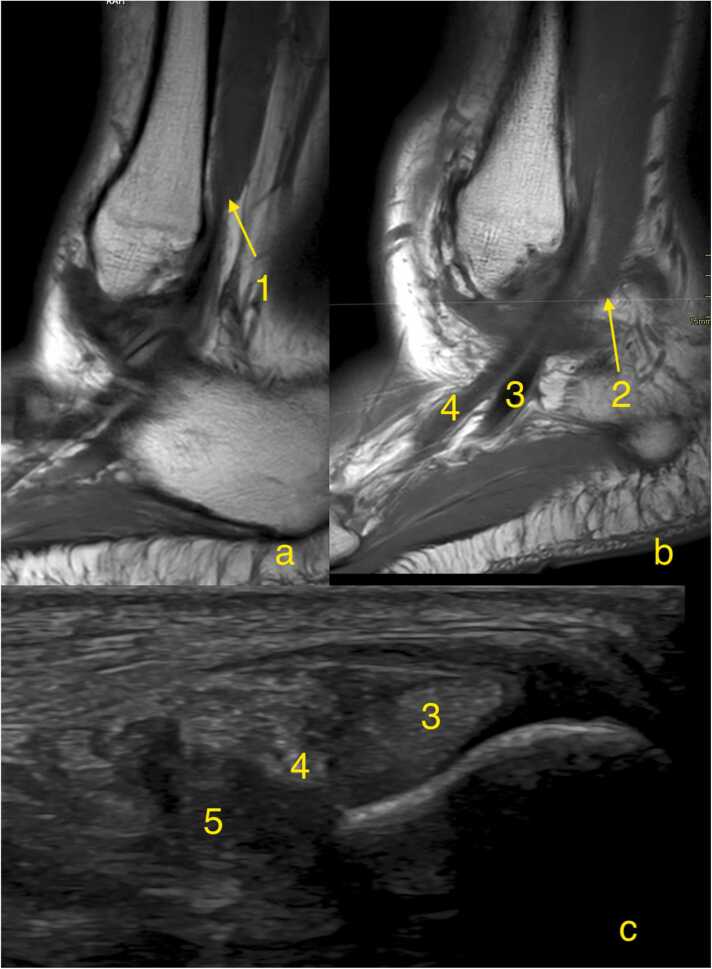
Anatomical variations of the peroneus brevis muscle in three different patients. (a, b) Sagittal cross section, T1-weighted images, (c) Transverse cross section ultrasound. 1- Muscle belly ending superior to the lateral malleolus, 2- Muscle belly ending at the tip of the lateral malleolus, considered a low-lying muscle belly, 3- Peroneus longus, 4- Peroneus brevis, 5- Muscle belly found in the superior peroneal tunnel, considered a low-lying muscle belly.

If the musculotendinous junction of peroneus brevis extends distal to the tip of the fibula, it is classified as a low-lying muscle belly (Fig. 6). A greater incidence of peroneus brevis split tears has been observed in cases with a low-lying peroneus brevis muscle belly[20]. However this variation can be seen also in asymptomatic volunteers[21]. During dorsiflexion, a more distal extension of the peroneus brevis muscle into the fibular groove can lead to stretching of the superior peroneal retinaculum, increasing pressure within the superior peroneal tunnel, which may contribute to split tears [3]. A low-lying muscle doesn't inherently lead to peroneal tendon pathology, but the overcrowding within superior peroneal tunnel may increase the risk of developing peroneal tendinopathy and split tear[14].

### 2.4 Accessory muscles (peroneus quartus and peroneus quintus)

**Fig. 7:**
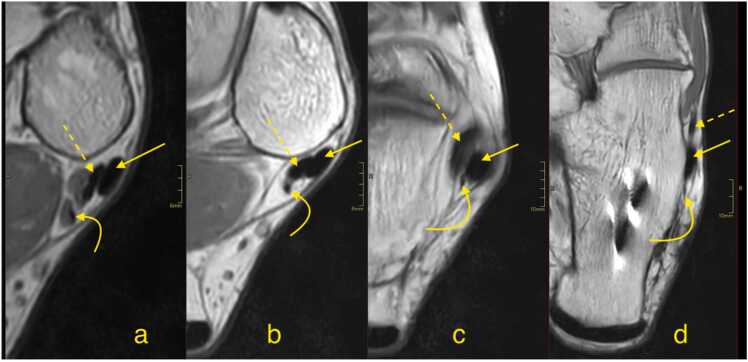
Proton density-weighted cross-sectional images showing the peroneus quartus (curved arrows) a-d. It runs posterior to the peroneus brevis (dashed arrow) and the peroneus longus (straight arrow). Metal artifacts are visible following calcaneus osteotomy on segment d.

The term "accessory peroneal muscle" typically refers to two muscles: the peroneus quartus and the peroneus digit quinti, with a prevalence of about 15 %, where the peroneus quartus accounts for about 10 % and the peroneus digit quinti for about 35 %[22], [23], [24]. The peroneus quartus (Fig. 7) and peroneus quintus can originate from the peroneus brevis, peroneus longus, fibula, or peroneus tertius. Their insertion points differ: the peroneus quartus inserts on the extensor digitorum longus slip or the retro trochlear tubercle of the calcaneus, while the peroneus quintus inserts on the dorsal aspect of the fifth metatarsal.

The presence of accessory muscles can cause pain and swelling around the lateral malleolus, likely due to attrition, overcrowding, and increased pressure within the superior peroneal tunnel[3], [8]. Some literature suggests a link to other pathologies, such as tendon split tear and instability, however this association remains unclear[8], [10].

The term “retromalleolar attrition syndrome” was first developed to describe tendon lesions in the peroneus brevis muscle, observed in a significant percentage of patients with a peroneus quartus muscle[25]. This condition is linked to the “overcrowding effect,” where the presence of an additional anatomical structure, such as the peroneus quartus, reduces the space within the peroneal sheath and increases the pressure in superior peroneal tunnel, leading to tendon compression[26]. The concept of “lateral ankle stenosis syndrome” was introduced as the last one to further explain how the peroneus quartus within the peroneal sheath can exacerbate this overcrowding, resulting in reduced space for the proper functioning of the peroneal tendons or even compressing them[23].

### 2.5 Peroneal tubercle

The peroneal tubercle is located below the lateral malleolus on the lateral surface of the calcaneus in bifurcation of the common peroneal synovial sheath. The peroneus brevis tendon passes superior to the peroneal tubercle, while the peroneus longus tendon passes inferiorly (Fig. 4). In our observations, a larger or smaller tubercle is usually visible in this location; however, in some cases, the tubercle may be absent, and the surface appears almost perfectly smooth. The peroneal tubercle is present in ca 84 % of symptomatic ankles[3] and its size varies but is usually less than 5 mm. Some authors use 5 mm as the cut-off for a hypertrophied peroneal tubercle, while others suggest that a higher threshold may be more appropriate to avoid overdiagnosis[27]. Cadaveric studies have shown that it is prominent in 29 % of cases[28]. There is some controversy regarding definition of the peroneal tubercle hypertrophy. In clinical practice, hypertrophy is often considered when the length of the peroneal tubercle exceeds the thickness of the peroneal tendons. When the peroneus brevis and peroneus longus tendons pass over a hypertrophied tubercle, mechanical irritation can occur. However, not all individuals with hypertrophy are symptomatic, so this finding should be interpreted with caution. Subsequent conditions such as tendonitis, stenosis, and tendon tears may arise as a result[2], [5], [29].

### 2.6 Interconnections in the lateral malleolus region

**Fig. 8:**
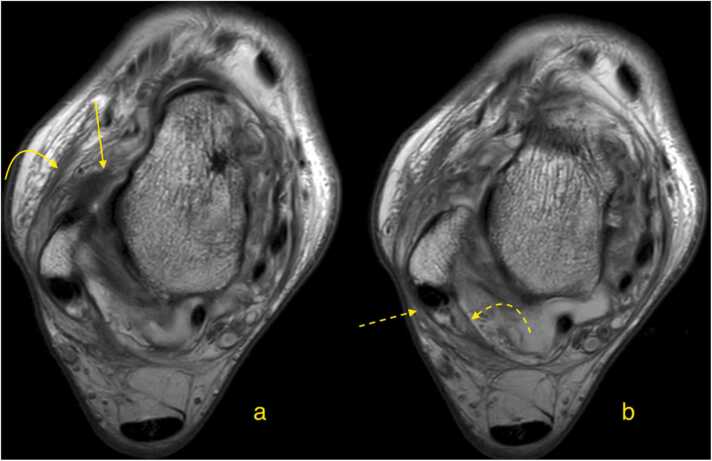
Patient after a sprain injury to the ankle. Magnetic resonance imaging (a and b) proton density-weighted, axial cross sections. The imaging revealed a complete rupture of the anterior talofibular ligament (straight arrow). Thickening of the crural fascia at the level of the lower part of the extensor retinaculum (curved arrow) is observed, along with thickening of the crural fascia at the level of the posterior boundary of the peroneal tunnel.

The ligaments and retinacula surrounding the ankle joint are interconnected, offering an anatomical explanation for why isolated injuries to a single structure in the ankle are relatively uncommon[30], [31]. The superior peroneal retinaculum connects anteriorly with the anterior talofibular ligament and the extensor retinaculum, and posteriorly with the leg's fascial system and the paratenon of the Achilles tendon[32]. The clinical significance of these connections is unclear. The presence of interconnections through fascial extensions may partially explain why ankle injuries frequently involve multiple structures (Fig. 8). Clinically observed concomitant injuries of the anterior talofibular ligament and the superior peroneal retinaculum may have a biomechanical basis, but the presence of interconnections could also contribute

## 3 Summary

When evaluating patients with suspicion of peroneus brevis split tear, it is crucial to assess the retromalleolar groove, superior peroneal retinaculum. Anatomical variations of the structures that form and pass through the superior peroneal tunnel may affect peroneal tendon’s function. The presence of a low-lying peroneus brevis or accessory tendons within the superior peroneal tunnel may increase pressure, contributing to tendinosis or tendon split tear. However, while anatomical variants may play a role in the development of peroneal tendon pathologies, they do not always lead to injury. This review highlights the importance of understanding the anatomy and its variations to improve diagnostic accuracy for peroneal tendon pathologies.

## Ethics approval and consent to participate

No ethics approval is needed for this pictorial review. Not applicable for pictorial review.

## Consent for publication

Not applicable. The manuscript does not contain the data of individuals in any form.

## Authors' contributions

PS conceived the idea of this review. KBD, RZ, DM, DD and PS contribute the first draft of the manuscript. KBD, DD and PS selected appropriate figures and prepared figures. All authors read and approved the final manuscript.

## Funding

We acknowledge the financial support of the 10.13039/100010819Tornspiran Foundation, which enabled the preparation of this review article. This work was completed as part of our broader research activities supported by the foundation. The foundation had no influence on the content or conclusions presented in this article.

## CRediT authorship contribution statement

**Katarzyna Bokwa-Dąbrowska:** Writing – review & editing, Writing – original draft, Methodology, Data curation, Conceptualization. **Rafał Zych:** Writing – review & editing, Writing – original draft, Visualization, Methodology. **Dan Mocanu:** Writing – review & editing, Writing – original draft, Visualization, Validation, Methodology. **Michael Huuskonen:** Writing – review & editing, Writing – original draft, Visualization. **Pawel Szaro:** Writing – review & editing, Writing – original draft, Visualization, Supervision, Methodology, Conceptualization. **Dawid Dziedzic:** Visualization, Writing – original draft, Writing – review & editing.

## Declaration of Generative AI and AI-assisted technologies in the writing process

Nothing to disclose

## Declaration of Competing Interest

The authors declare that they have no known competing financial interests or personal relationships that could have appeared to influence the work reported in this paper.

## Data Availability

Yes.

## References

[1] Galli M.M., Protzman N.M., Mandelker E.M., Malhotra A.D., Schwartz E., Brigido S.A. (2015). An examination of anatomic variants and incidental peroneal tendon pathologic features: a comprehensive MRI review of asymptomatic lateral ankles. J. Foot Ankle Surg..

[2] Squires N., Myerson M.S., Gamba C. (2007). Surgical treatment of peroneal tendon tears. Foot Ankle Clin..

[3] Ersoz E., Tokgoz N., Kaptan A.Y., Ozturk A.M., Ucar M. (2019). Anatomical variations related to pathological conditions of the peroneal tendon: evaluation of ankle MRI with a 3D SPACE sequence in symptomatic patients. Skelet. Radio..

[4] Taparia A., Kumar S., Saran S. (2022). Lateral retromalleolar swelling and pain - peroneus brevis tendon tear (Boomerang Sign). J. Med Ultrasound.

[5] Wang X.T., Rosenberg Z.S., Mechlin M.B., Schweitzer M.E. (2005). Normal variants and diseases of the peroneal tendons and superior peroneal retinaculum: MR imaging features. Radiographics.

[6] Rademaker J., Rosenberg Z.S., Delfaut E.M., Cheung Y.Y., Schweitzer M.E. (2000). Tear of the peroneus longus tendon: MR imaging features in nine patients. Radiology.

[7] Drakonaki E.E., Gataa K.G., Solidakis N., Szaro P. (2021). Anatomical variations and interconnections of the superior peroneal retinaculum to adjacent lateral ankle structures: a preliminary imaging anatomy study. J. Ultrason.

[8] Athavale S.A., Swathi, Vangara S.V. (2011). Anatomy of the superior peroneal tunnel. J. Bone Jt. Surg. Am..

[9] Schubert R. (2013). MRI of peroneal tendinopathies resulting from trauma or overuse. Br. J. Radio..

[10] Davda K., Malhotra K., O'Donnell P., Singh D., Cullen N. (2017). Peroneal tendon disorders. EFORT Open Rev..

[11] Kudoh H., Sakai T., Horiguchi M. (1999). The consistent presence of the human accessory deep peroneal nerve. J. Anat..

[12] Sobel M., Geppert M.J., Warren R.F. (1993). Chronic ankle instability as a cause of peroneal tendon injury. Clin. Orthop. Relat. Res.

[13] Mirmiran R., Squire C., Wassell D. (2015). Prevalence and role of a low-lying peroneus brevis muscle belly in patients with peroneal tendon pathologic features: a potential source of tendon subluxation. J. Foot Ankle Surg..

[14] van Dijk P.A., Miller D., Calder J., DiGiovanni C.W., Kennedy J.G., Kerkhoffs G.M., Kynsburtg A., Havercamp D., Guillo S., Oliva X.M., Pearce C.J., Pereira H., Spennacchio P., Stephen J.M., van Dijk C.N. (2018). The ESSKA-AFAS international consensus statement on peroneal tendon pathologies. Knee Surg. Sports Trauma. Arthrosc..

[15] Kumai T., Benjamin M. (2003). The histological structure of the malleolar groove of the fibula in man: its direct bearing on the displacement of peroneal tendons and their surgical repair. J. Anat..

[16] Ozbag D., Gumusalan Y., Uzel M., Cetinus E. (2008). Morphometrical features of the human malleolar groove. Foot Ankle Int.

[17] Adachi N., Fukuhara K., Kobayashi T., Nakasa T., Ochi M. (2009). Morphologic variations of the fibular malleolar groove with recurrent dislocation of the peroneal tendons. Foot Ankle Int.

[18] Mabit C., Salanne P., Blanchard F., Boncoeur-Martel M.P., Fiorenza F. (1999). The retromalleolar groove of the fibula: a radio-anatomical study. Foot Ankle Surg..

[19] Patil M., Kulkarni M.S., Sinha A., Ghorpade R.R. (2024). Biomechanical variations in patients with flatfoot deformity: Impact of gender, age, and BMI on foot kinetics and kinematics. J. Orthop..

[20] Freccero D.M., Berkowitz M.J. (2006). The relationship between tears of the peroneus brevis tendon and the distal extent of its muscle belly: an MRI study. Foot Ankle Int.

[21] Saupe N., Mengiardi B., Pfirrmann C.W., Vienne P., Seifert B., Zanetti M. (2007). Anatomic variants associated with peroneal tendon disorders: MR imaging findings in volunteers with asymptomatic ankles. Radiology.

[22] Yammine K. (2015). The accessory peroneal (fibular) muscles: peroneus quartus and peroneus digiti quinti. A systematic review and meta-analysis. Surg. Radio. Anat..

[23] Donley B.G., Leyes M. (2001). Peroneus quartus muscle. A rare cause of chronic lateral ankle pain. Am. J. Sports Med.

[24] Sobel M., Levy M.E., Bohne W.H. (1990). Congenital variations of the peroneus quartus muscle: an anatomic study. Foot Ankle.

[25] Sobel M., Geppert M.J., Olson E.J., Bohne W.H., Arnoczky S.P. (1992). The dynamics of peroneus brevis tendon splits: a proposed mechanism, technique of diagnosis, and classification of injury. Foot Ankle.

[26] White A.A., 3rd, Johnson D., Griswold D.M. (1974). Chronic ankle pain associated with the peroneus accessorius. Clin. Orthop. Relat. Res.

[27] Vosoughi A.R., Tabatabaei M. (2021). CT scan assessment of the dimensions and morphological variations of the peroneal tubercle. Foot Ankle Surg..

[28] Hyer C.F., Dawson J.M., Philbin T.M., Berlet G.C., Lee T.H. (2005). The peroneal tubercle: description, classification, and relevance to peroneus longus tendon pathology. Foot Ankle Int.

[29] Sharma A., Parekh S.G. (2020). Pathologies of the Peroneals: A Review. Foot Ankle Spec..

[30] Dalmau-Pastor M., Malagelada F., Calder J., Manzanares M.C., Vega J. (2020). The lateral ankle ligaments are interconnected: the medial connecting fibres between the anterior talofibular, calcaneofibular and posterior talofibular ligaments. Knee Surg. Sports Trauma. Arthrosc..

[31] Szaro P., Ghali Gataa K., Polaczek M., Ciszek B. (2020). The double fascicular variations of the anterior talofibular ligament and the calcaneofibular ligament correlate with interconnections between lateral ankle structures revealed on magnetic resonance imaging. Sci. Rep..

[32] Szaro P., Polaczek M., Ciszek B. (2020). The Kager's fat pad radiological anatomy revised. Surg. Radio. Anat..

